# Syncytiotrophoblast extracellular microvesicle profiles in maternal circulation for noninvasive diagnosis of preeclampsia

**DOI:** 10.1038/s41598-020-62193-7

**Published:** 2020-04-14

**Authors:** Lisa Levine, Andreas Habertheuer, Chirag Ram, Laxminarayana Korutla, Nadav Schwartz, Robert W. Hu, Sanjana Reddy, Andrew Freas, Patrick D. Zielinski, Joey Harmon, Sudheer Kumar Molugu, Samuel Parry, Prashanth Vallabhajosyula

**Affiliations:** 1 Division of Maternal Fetal Medicine, Department of Obstetrics and Gynecology, University of Pennsylvania, Pennsylvania, USA; 2 Division of Cardiovascular Surgery, Department of Surgery, University of Pennsylvania, Pennsylvania, USA; 3 Department of Biochemistry and Biophysics, University of Pennsylvania, Pennsylvania, USA; 4 Division of Cardiac Surgery, Department of Surgery, Yale University School of Medicine, New Haven, USA

**Keywords:** Diagnostic markers, Reproductive disorders

## Abstract

Preeclampsia is the most common placental pathology in pregnant females, with increased morbidity and mortality incurred on the mother and the fetus. There is a need for improved biomarkers for diagnosis and monitoring of this condition. Placental syncytiotrophoblasts at the maternal-fetal interface release nanoparticles, including extracellular microvesicles, into the maternal blood during pregnancy. Syncytiotrophoblast extracellular microvesicles (STEVs) are being studied for their diagnostic potential and for their potential physiologic role in preeclampsia. We hypothesized that STEV profiles in maternal circulation would be altered under conditions of preeclampsia compared to normal pregnancy. Extracellular vesicles (EVs) released by BeWo cells *in vitro* showed high expression of syncytin-1, but no plac1 expression, demonstrating that trophoblast cell EVs express syncytin-1 on their surface. Placental alkaline phosphatase also showed high expression on BeWo EVs, but due to concern for cross reactivity to highly prevalent isoforms of intestinal and bone alkaline phosphatase, we utilized syncytin-1 as a marker for STEVs. *In vivo*, syncytin-1 protein expression was confirmed in maternal plasma EVs from Control and Preeclampsia subjects by Western blot, and overall, lower expression was noted in samples from patients with preeclampsia (n = 8). By nanoparticle analysis, EV profiles from Control and Preeclampsia groups showed similar total plasma EV quantities (p = 0.313) and size distribution (p = 0.415), but STEV quantitative signal, marked by syncytin-1 specific EVs, was significantly decreased in the Preeclampsia group (p = 2.8 × 10^−11^). Receiver operating characteristic curve demonstrated that STEV signal threshold cut-off of <0.316 was 95.2% sensitive and 95.6% specific for diagnosis of preeclampsia in this cohort (area under curve = 0.975 ± 0.020). In conclusion, we report that the syncytin-1 expressing EV profiles in maternal plasma might serve as a placental tissue specific biomarker for preeclampsia.

## Introduction

Preeclampsia is the most common pregnancy-associated disorder, affecting up to 3 to 5% of pregnancies^1–4^. In addition to increased morbidity and mortality seen in the mother, preeclampsia is also associated with fetal complications, including premature birth and fetal growth restriction. Preeclampsia manifests as new onset hypertension (systolic blood pressure >140 mmHg and diastolic pressure >90 mmHg; proteinuria), which can occur at an early stage or late stage during pregnancy. Its severity can also vary and is classified as mild, moderate, severe preeclampsia, and eclampsia^5–7^. The precise mechanistic cause of preeclampsia is being extensively investigated, but there is wide agreement that the disorder originates in the placenta^8–14^, and is related to decreased uteroplacental blood flow^8,9,11–15^, which leads to a cascade of physiologic processes manifesting clinically as a pregnancy-associated hypertensive disorder^16–18^.

Placental trophoblasts, especially syncytiotrophoblasts, are fetal-derived cells that lie at the maternal-fetal interface and tightly regulate exchange of nutrients, metabolites, and other macromolecules between these two entities. Several studies have shown that syncytiotrophoblasts are highly active in fetal physiology, and their dysfunction has been implicated in placental pathophysiology^19^. In addition, syncytiotrophoblasts also release a wide array of extracellular vesicles into the maternal circulation throughout gestation^19–23^, with increasing amounts seen during the latter stages of pregnancy. These include apoptotic bodies, microvesicles such as ectosomes and microparticles, and extracellular microvesicles (EVs) including exosomes, which are released via different mechanistic processes and are detectable in peripheral blood as microvesicles of different sizes. Although the exact definition of these microvesicles by size remains somewhat nebulous, there is excellent consensus that exosomes are nanoparticles in the range of 30 to 150 nm that are specifically released through endosomal pathway utilizing endocytic machinery via subcellular structures called multivesicular bodies^24^. Exosomes express canonical and tissue specific proteins on their surface, and their intraexosomal compartments are enriched in functional macromolecules implicated to have paracrine and systemic effects on target tissues^24,25^. Recently, several groups have investigated the diagnostic and physiologic implications of maternal plasma microvesicles, including EVs, in normal pregnancy and in pregnancy-associated disorders. These studies suggest that in addition to changes in total maternal plasma microvesicle pools, selective expression levels of circulating placental proteins may also be altered in conditions of normal pregnancy versus pregnancy-associated disorders such as preeclampsia. In this context, three placental proteins, placental alkaline phosphatase (PLAP), syncytin-1, and syncytin-2 have been studied^19,26–33^. These studies showed alterations in placental protein levels in EVs isolated from maternal plasma samples from subjects with preeclampsia versus normal pregnancy, although with some mixed results. But overall, the studies validate the concept that placental dysfunction is associated with altered circulating placental microvesicles/ placental proteins in maternal plasma, and therefore, characterization of their profiles has diagnostic potential for noninvasive detection and monitoring of pregnancy-associated disorders.

Recently, our group investigated the diagnostic potential of tissue specific EV profiling as a noninvasive diagnostic for early detection and monitoring of immunologic rejection in transplantation^34,35^. We demonstrated that injury to the transplant tissue leads to early changes in transplant tissue specific EVs in recipient blood, and their profiles predicted early acute rejection with high accuracy in a time-sensitive manner. We translated this concept in the setting of pregnancy-associated disorders and hypothesized that placental injury/ dysfunction associated with preeclampsia might lead to alterations in placental-specific EVs in maternal plasma. If so, as suggested by other groups, characterization of placenta-specific EVs might serve as a novel, diagnostic biomarker for preeclampsia. We report our initial investigation of this concept.

## Materials and Methods

### Study design and subject samples

This was a planned sub-study utilizing blood samples that were collected from women enrolled in a prospective longitudinal study evaluating biomarker differences in women with and without severe preeclampsia. Women were enrolled in this study from April 2015 to May 2017 at the Hospital of the University of Pennsylvania after obtaining written informed consent. Institutional Review Board approval was obtained prior to initiation of the study (IRB #821592). We also confirm that all experiments were performed in accordance with the relevant guidelines and regulations. Cases were women diagnosed with preterm (23–36 6/7 weeks) preeclampsia with severe features who were admitted to the Obstetrical unit at our hospital. Preeclampsia with severe features was defined by current guidelines from the Hypertension Task Force of the American College of Obstetricians and Gynecologists^36^. Normotensive controls were recruited in the outpatient setting and matched by gestational age of preeclampsia diagnosis (±3 weeks), race, maternal age (±8 years), and body mass index (±5 kg/m^2^). Blood was drawn at the time of enrollment for both cases and controls. Women were followed prospectively into the postpartum period. Controls who developed any form of pregnancy related hypertension were subsequently excluded post enrollment. All women with preexisting cardiovascular disease were excluded. Baseline patient demographics and characteristics are summarized in Table 1.

**Table 1:** Demographics and patient characteristics.

	Case (n = 21)	Control (n = 23)	P value
Maternal age – mean ± SD (years)	29.7 ± 8.2	26.4 ± 5.1	0.12
Race – n (%)			
African American	19 (90.5)	20 (87.0)	0.71
White	2 (9.5)	3 (13.0)	
Body Mass Index - mean ± SD (kg/m^2^)	31.2 ± 7.5	25.6 ± 6.2	0.01
Gestational age at blood draw* - mean ± SD (weeks)	31.3 ± 4.4	33.2 ± 4.0	0.63
Parity – median [IQR]	0 [0–1]	1 [0–1]	0.46
Tobacco use – n (%)	0 (0)	2 (8.7)	0.43
Chronic HTN – n (%)	12 (57.1)	1 (4.3)	0.02
**Postnatal outcomes^**			
Gestational age at delivery – median [IQR] (weeks)	32 [29–36]	39 [38–40]	P < 0.001
Birth weight (g) - mean ± SD	1720 ± 993	3343 ± 641	P < 0.001

*Equates to the gestational age of onset of preeclampsia for the cases.

^By definition, all cases were delivered preterm.

### BeWo cell line and EV isolation

To validate the presence of syncytiotrophoblast specific markers on EV surface, we first analyzed for these markers in an *in vitro* system. Human choriocarcinoma-derived cell line (BeWo) was grown in Dulbecco’s modified Eagle’s medium (DMEM) (with L-glutamine and 4500 mg glucose/L, without sodium bicarbonate; Sigma Chemical Co., Missouri, cat. no. D-5648), heat-inactivated fetal bovine serum (FBS) (Atlanta Biologicals, Georgia) and penicillin/streptomycin (10,000 U/mL; Invitrogen, California) until 90% confluent. Culture medium was replaced with Exosome free FBS and cells were grown for another 48 hours. Culture medium was collected and EVs were isolated using methodology of ultracentrifugation. First, culture medium was spun at 800 g for 5 minutes followed by 2000 g for 10 minutes to remove cellular debris. Supernatant was collected and ultrafiltered using a 100 kDa cut-off membrane, followed by ultracentrifugation at 120,000 g for 2 hours at 4 °C. Pellet containing EVs were resuspended in 1 × phosphate buffered saline (PBS)

### Plasma EV isolation

Blood was collected from all patients in EDTA tubes and was immediately spun down at 2,000 g for 15 minutes at 4 °C. Plasma was removed and aliquoted into 250 µL portions. The plasma was frozen at −80 °C until all samples were collected in order to isolate all EVs at the same time. 250 µL of human plasma was passed through size exclusion chromatography column to obtain eluent fractions containing EVs^35^. The pooled fractions were filtered through a 100 kDa cut-off membrane (Thermo Fisher Scientific, Waltham, MA) and ultracentrifuged at 120,000 g for 2 hours at 4 °C. The spun-down pellet containing EVs was resuspended in 400 μL of phosphate buffered saline (1xPBS) for downstream analysis.

### Cryo-electronmicroscopy

Extracellular vesicles (3 ul) were applied onto 200-mesh copper grids (Quantifoil R1.2/1.3) that were glow discharged for 60 seconds. Excess solution was blotted with filter paper for 5.5 Sec using Vitrobot Mark IV (FEI Netherlands) at 4 °C and the grids were immediately flash frozen by rapidly plunging the grid into liquid ethane at −165 °C. CryoEM data for the sample was collected on a FEI Tecnai F 200 KeV TEM microscope operated at 200 keV. Images were recorded on Falcon III direct electron detector at a magnification of 25,000x. The CryoEM micrograph was generated by averaging individual dose fractionated frames collected at a rate of 40 frames/second for 4 second exposures. The frames were motion corrected and summed into a single micrograph.

### Nanoparticle detector analysis

Isolated EVs from maternal plasma and BeWo cell line EVs were analyzed on the NanoSight NS300 nanoparticle detector light scatter mode (Malvern Instruments Inc., Massachusetts) for quantitation and size distribution of EVs. All captures were taken at a camera level of 16 with a detection threshold of 10. For STEV subpopulation analysis, EVs were studied for surface expression of synctin-1, PLAP, and PLAC-1 using anti-human syncytin-1 (Santa Cruz Biotechnology, California), anti-PLAP (Santa Cruz Biotechnology, California) and anti-PLAC-1 (Santa Cruz Biotechnology, California) antibody conjugated quantum dots (Thermo Fisher Scientific, Massachusetts) on the nanoparticle detector fluorescence mode. Rabbit IgG, mouse IgG and goat IgG antibody quantum dot (Santa Cruz, California) were used as isotype controls. Each sample was run in duplicates and each experimental run was duplicated independently; the mean value of the two independent runs is represented. In each displayed panel, the nanoparticle size distribution curve is represented by particle size (nanometers) on the x-axis and nanoparticle concentration (x10^6^/ml) on the y-axis. The curve in blue represents total plasma EV pool distribution, and red curve represents the respective subpopulation.

### Western blot analysis

BeWo cells were lysed using radioimmunoprecipitation assay (RIPA) buffer (Thermo Fisher Scientific, Massachusetts). Cell lysates were used as a positive control and as tissue confirmation of the protein markers being assessed. Equal quantities of EV protein and cell lysate (10 µg) were run on polyacrylamide gels and then transferred onto nitrocellulose membranes (Life Technologies, New York). After blocking with 5% milk, membranes were probed with primary antibodies specific to the following proteins: syncytin-1 (1:500, Santa Cruz Biotechnology, California), PLAP (1:200, Santa Cruz Biotechnology, California), Plac-1 (1:200, Santa Cruz Biotechnology, California), CD63 (1:1000, Santa Cruz Biotechnology, California), Cytochrome C (1:500, Santa Cruz Biotechnology, California), β-actin (1:200, Proteintech, Illinois), and flotillin-1 (1:1000, Proteintech, Illinois). Horseradish peroxidase coupled secondary antibodies were added and detected through chemiluminescence using ImageQuant LAS 400 phosphoimager.

### RNA isolation and mRNA expression using reverse transcription-PCR

Total RNA was extracted from EVs using Trizol, followed by RNeasy mini kit, according to manufacturer’s protocol (Qiagen, Germany). Total RNA (25 to 50 ng) from EVs were reverse transcribed with the SuperScript III one-step RT-PCR system (Life Technologies) for syncytin-1 and β-actin gene expression.

### Statistical analysis

First, data were checked for distribution. Comparative analysis for continuous variables (total EV concentration and STEV signal for Preeclampsia versus healthy pregnant control) were performed using independent sample t-test (2-tailed).

For receiver operating characteristic (ROC) curve, the true-positive rate (sensitivity) was plotted against the false-positive rate (specificity) to illustrate performance of a binary classifying system (Control versus Preeclampsia groups). A threshold was determined using the Youden index, and likelihood ratio, sensitivity and specificity were calculated. ROC curves were compared using the method of Delong *et al*.^37^. General statistics were assessed using StataMP version 14.2 (StataCorp LP, Texas), and scatter plots and NanoSight panels were constructed using Prism version 7.0 (GraphPad, California). All reported tests were 2-tailed and alpha level was set to 0.05.

## Results

### EVs released by BeWo cells express placenta specific proteins

**Figure 1:**
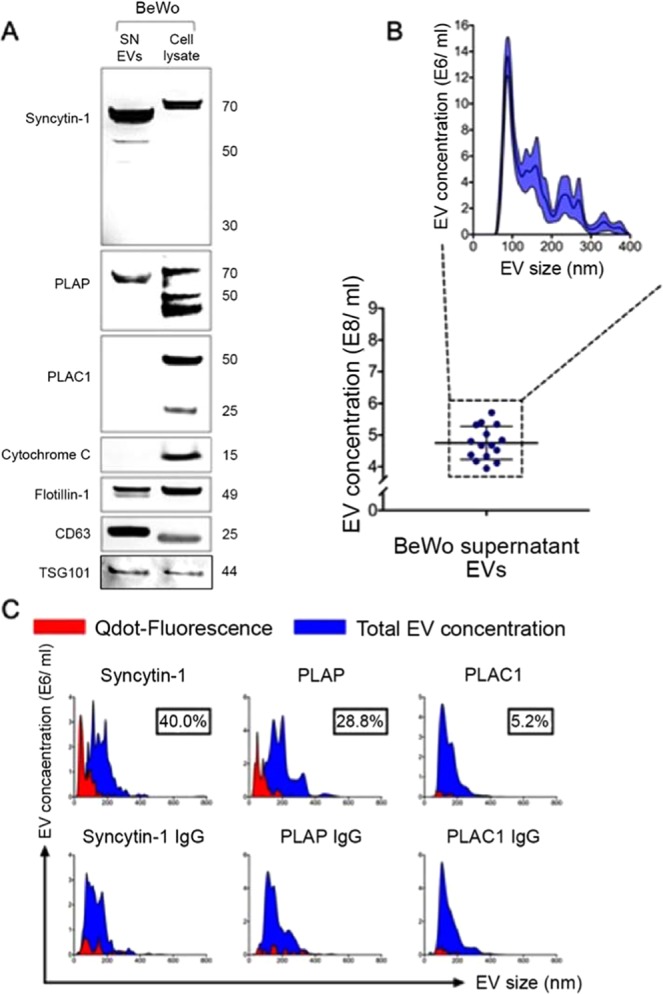
EVs released by BeWo cells express placenta specific protein markers. (**A**) Western blot analysis of EVs released into culture supernatant from BeWo cells. Supernatant EVs were positive for canonical exosomal markers CD63 and flotillin-1, suggesting that EVs isolated utilizing methodology detailed yielded nanoparticles. There was no contamination from cellular debris and apoptotic bodies in EV fractions (cytochrome C). BeWo EVs showed high expression of placental proteins syncytin-1 and PLAP, but not of PLAC-1. The blots were cropped from different gels. **(B)** Nanoparticle scatter analysis of BeWo EV preparations showed similar size distribution, with majority of EVs in the size range of 50–100 nm along with lower concentrations of microvesicles of larger sizes. **(C)** NanoSight nanoparticle detector analysis for surface expression of placental proteins is shown. Higher abundance of placenta-specific markers syncytin-1 and PLAP was noted compared to PLAC-1, which was not elevated compared to IgG isotype background fluorescence. EV subpopulations positive for syncytin-1, PLAP, and PLAC-1 (red) are shown in relation to the total EV pool (blue). Appropriate IgG isotypes (mouse IgG, rabbit IgG) were used as negative controls.

EV fraction obtained from BeWo culture supernatant was confirmed by Western blot for expression of canonical exosome markers flotillin-1, CD63, and for minimal contamination with other microvesicles such as apoptotic bodies (cytochrome C) (Fig. 1A). We then looked for expression of 3 placenta-specific proteins, syncytin-1, PLAP, and plac-1. BeWo EVs showed high expression of syncytin-1 and PLAP, but no expression of plac-1 (Fig. 1A). Nanoparticle analysis of EV preparations of supernatant samples from different BeWo cell cultures showed similar size distribution, with majority of the EVs in the size range consistent with exosomes (<200 nm) (Fig. 1B). EVs were next studied on the nanoparticle detector for surface expression of placental proteins (Fig. 1C). Syncytin-1 and PLAP expression was noted at high levels, compared to plac-1. Taken together, these studies suggest that trophoblast cells release EVs that express placental proteins on their surface. Given the literature supporting expression of PLAP and syncytin-1 in maternal plasma during pregnancy, we chose to further study the utility of syncytin-1 as a diagnostic marker for syncytiotrophoblast EVs in maternal plasma.

### Patient clinical data

Demographics, clinical parameters, and neonatal data are shown in Table 1. Subjects with preeclampsia were older (29.7 ± 8.2 years versus 26.4 ± 5.1 years; p = 0.12) and had higher body mass index (31.2 ± 7.5 kg/m^2^ versus 25.6 ± 6.2 kg/m^2^; p = 0.01).

### Maternal plasma EV analysis

**Figure 2:**
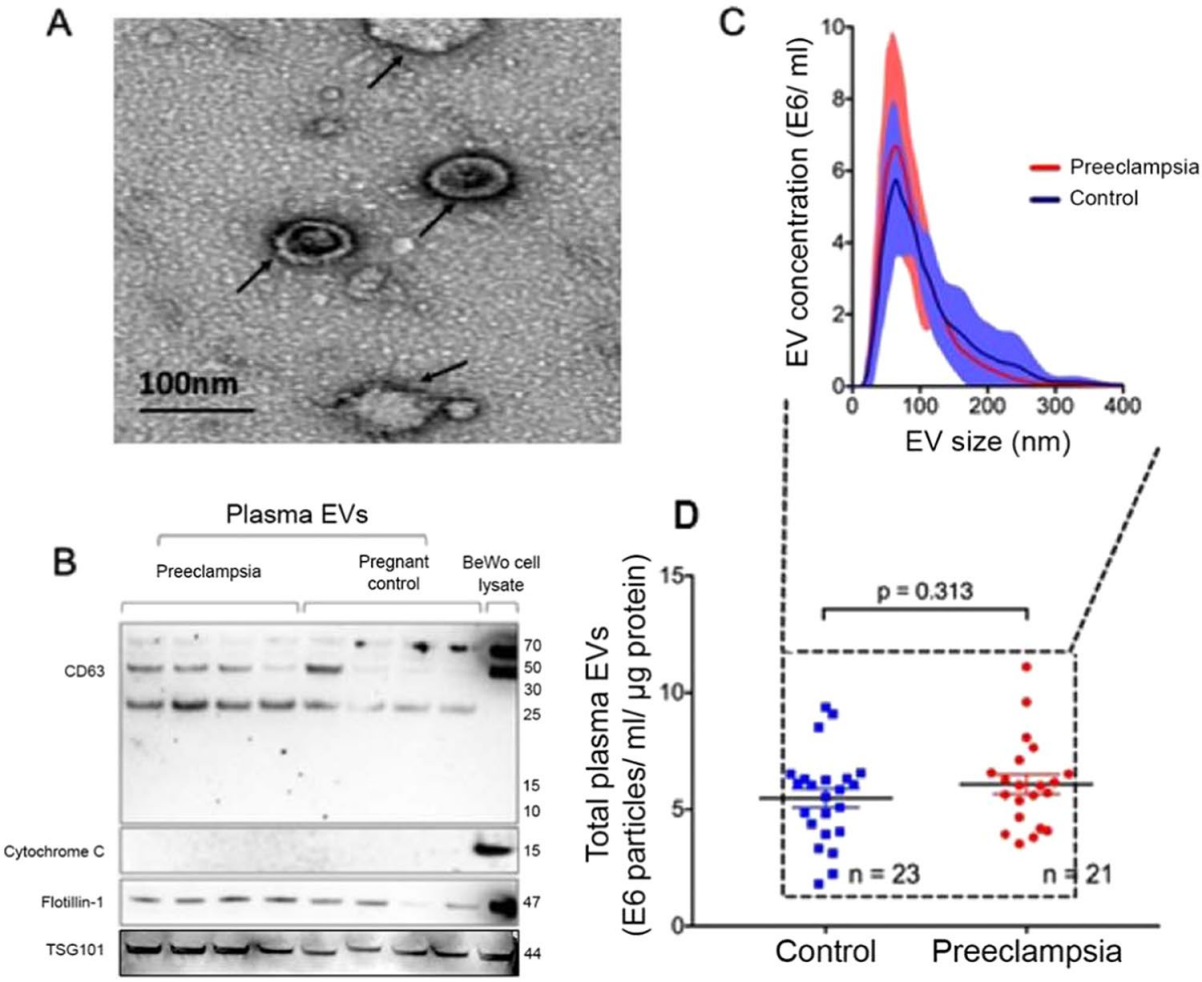
EVs isolated from maternal circulation express canonical exosome markers. (**A**) A representative of Cryo EM image of plasma EVs isolated from normal pregnant individual (scale bar 100 nm). **(B)** EVs isolated utilizing methodology detailed yielded nanoparticles enriched in exosome markers CD63, TSG101 and flotillin-1 without contamination from cellular debris and apoptotic bodies (cytochrome C). The blots were cropped from different parts of the same gel. **(C)** Nanoparticle scatter analysis confirmed that majority of isolated EVs had size distribution consistent with exosomes (50–100 nm), and there was no difference in the size distribution of EVs between the Preeclampsia versus Control groups (p = 0.415). **(D)** Total plasma EV quantities in Preeclampsia versus Control groups were also similar (p = 0.313).

First, morphology and size of the EVs were examined by cryo electron microscopy (Fig. 2A). Next, we confirmed that the EVs isolated using the methodology detailed expressed canonical exosome markers CD63 and flotillin-1 (Fig. 2B), with minimal contamination from cellular debris and apoptotic bodies. Further, nanoparticle analysis showed that the majority of isolated EVs had size distribution consistent with exosomes, with peak distribution at <100 nm (Fig. 2C). Next, we analyzed whether total EV quantities were altered in normal pregnancy versus preeclampsia. There was no statistical difference in total EV quantities in normal pregnancy controls versus preeclampsia subjects (p = 0.313, Fig. 2D).

**Figure 3:**
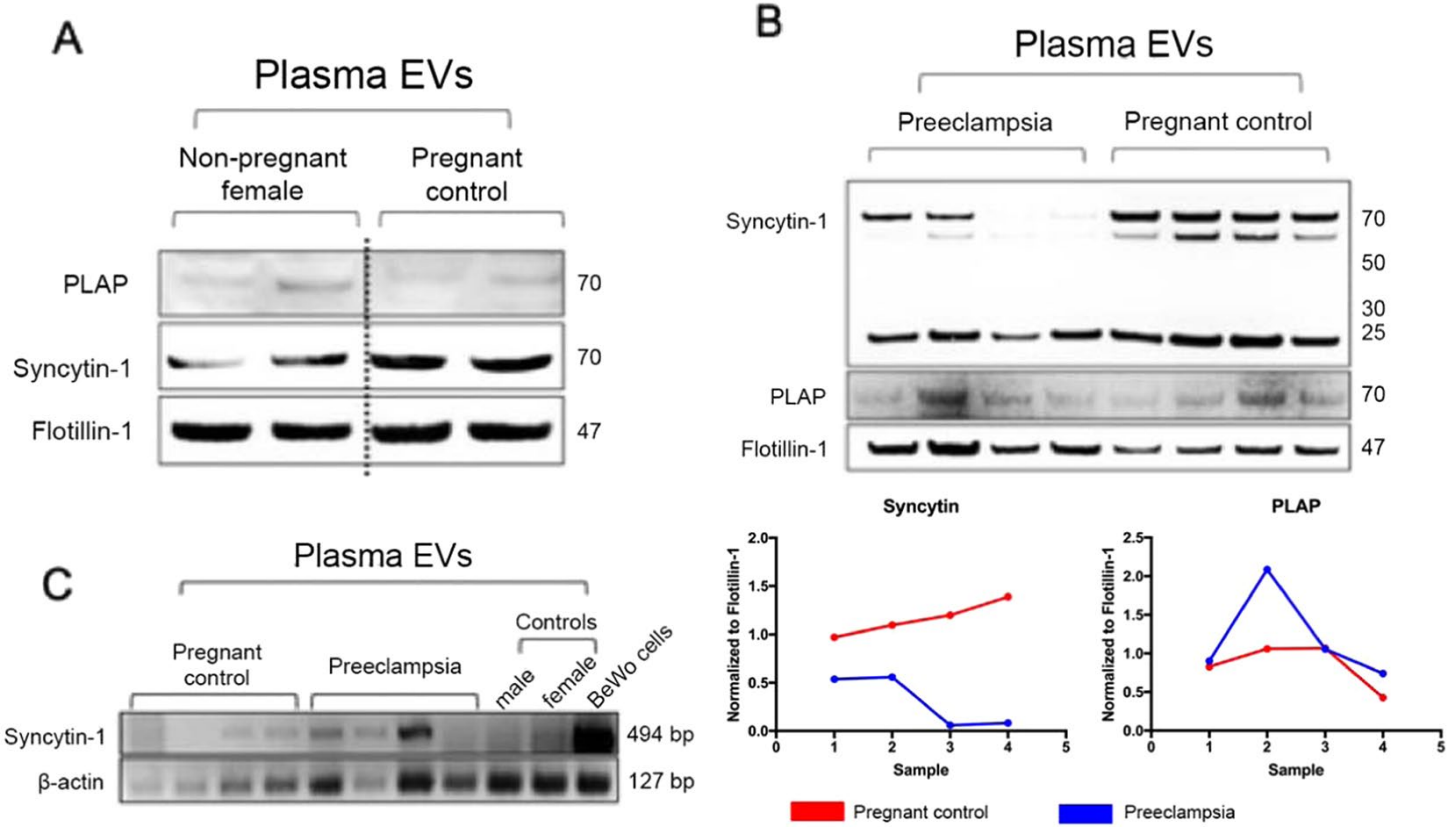
Syncytin-1 expression in preeclampsia patients compared to healthy pregnant controls. (**A**) EVs from women were analyzed for PLAP expression by Western Blot. Similar levels of PLAP were seen in non-pregnant female controls compared to pregnant women. Presence of flotillin-1 suggested that EVs isolated utilizing methodology detailed yielded nanoparticles enriched in exosomes. The blots were cropped from different gels. **(B)** Maternal plasma EV samples were analyzed for syncytiotrophoblast protein marker expression by Western blot. Similar levels of PLAP were seen in preeclampsia versus control samples. Lower levels of syncytin-1 were seen in preeclampsia subjects compared to healthy pregnant controls. Canonical exosome marker, flotillin-1, is shown as positive control. The blots were cropped from different parts of the same gel. Normalized values to flotillin-1 are represented for Syncytin-1 and PLAP for pregnant control (red) and preeclampsia subject (blue). **(C)** RT-PCR analysis of plasma EV mRNA showed similar levels of syncytin-1 in both preeclampsia and control subjects. The blots were cropped from different parts of the same gel.

Given these findings, next we assessed whether plasma EVs express syncytiotrophoblast protein markers. On Western blot, PLAP detection was noted in maternal plasma EV samples from Control and Preeclampsia groups. Furthermore, there was also detection of PLAP in non-pregnant females, suggesting that antibody was possibly cross reacting to alkaline phosphatase isoforms released by other tissue types (Fig. 3A). Next, we tested for syncytin-1 expression. Compared to male and non-pregnant female controls, which showed very low expression of syncytin-1, maternal plasma EVs from control subjects showed high syncytin-1 expression by Western blot (Fig. 3A). This difference was more pronounced with syncytin-1 than PLAP. In addition, subjects from the Preeclampsia group showed decreased syncytin-1 expression (Fig. 3B). Therefore, syncytin-1 levels in the plasma EV pools suggested decreased expression in preeclampsia subjects compared to pregnant controls.

Next, we assessed whether differential protein expression in the plasma EV pool observed translates to differences at the mRNA level. We observed a much more heterogeneous syncytin-1 mRNA expression compared to the protein level (Fig. 3C), and differential syncytin-1 protein expression could not be extrapolated to the mRNA level. Taken together, these data demonstrated that syncytin-1 protein expression is upregulated in the plasma EV pool during pregnancy, and preeclampsia leads to changes in its expression at the protein level, but possibly not at the mRNA level. Given the specificity of syncytin-1 expression in placental trophoblasts, these data suggest that preeclampsia is associated with decreased production or increased clearance of EVs released by syncytiotrophoblasts into maternal plasma.

### Quantitative analysis of syncytiotrophoblast EVs

**Figure 4:**
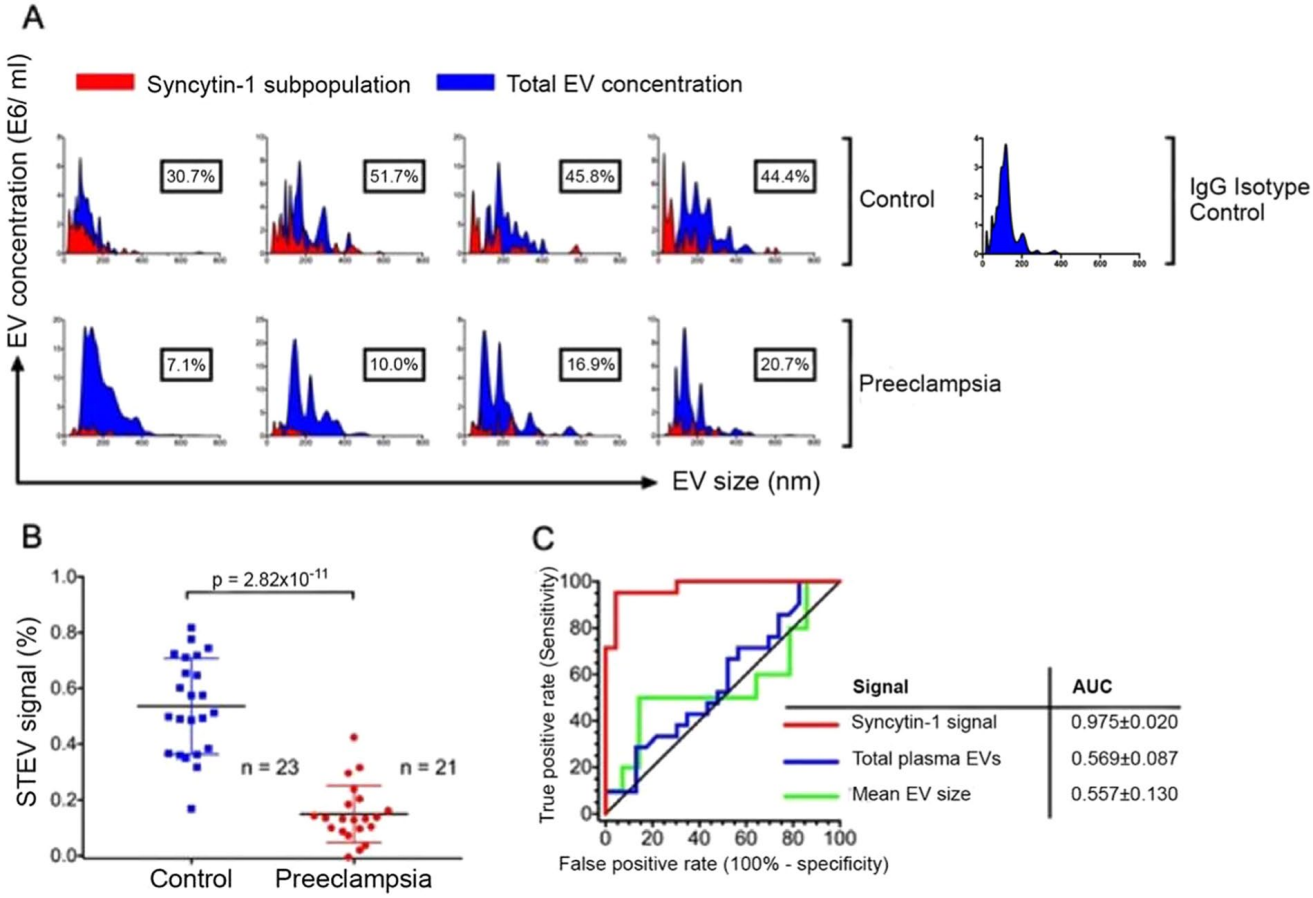
Decreased syncytin-1 EV signal in maternal circulation enables diagnosis of preeclampsia with high accuracy. (**A**) Syncytiotrophoblast EVs were quantified on the nanoparticle detector using anti-syncytin-1 antibody conjugated quantum dots. This demonstrated that preeclampsia subjects had lower levels of syncytin-1 expressing EVs compared to control subjects. NanoSight panels show syncytin-1 positive EV subpopulation (red) in relation to total plasma EV pool (blue). **(B)** Scatter plot analysis of STEV signal showed significantly decreased syncytin-1 signal in preeclampsia subjects (p = 2.82 × 10^−11^). **(C)** Receiver operating characteristic curve (ROC) in a binary cohort of preeclampsia versus control subjects was constructed for syncytin-1 EV signal, total plasma EVs quantity, and mean plasma EV size. This demonstrated an area under the curve of 0.975 ± 0.020 for STEV quantitative profiling, and a syncytin-1 EV signal threshold level of <0.316 predicted preeclampsia in this cohort with 95.2% sensitivity and 95.6% specificity. Total plasma EV numbers and mean plasma EV size had low diagnostic accuracy compared to STEV signal quantitation.

Although total EV levels were indifferent between groups, Western blot analyses suggested that decreased levels of syncytin-1 expressing syncytiotrophoblast EVs are seen with preeclampsia. Therefore, we quantified the syncytiotrophoblast EVs on the nanoparticle detector using anti-syncytin-1 antibody conjugated quantum dots. This demonstrated that Preeclampsia subjects had lower levels of syncytin-1 expressing EVs compared to control subjects (Fig. 4A). Syncytin-1 EV quantitative signal was significantly lower in the Preeclampsia group (Fig. 4B) in accordance to the Western blot findings. Given this difference, we generated a receiver operating characteristic curve to understand the diagnostic potential of sycytin-1 EV signal to distinguish between preeclampsia versus normal pregnancy. This demonstrated an area under the curve of 0.975 ± 0.020. Syncytin-1 EV signal threshold level of <0.316 predicted preeclampsia in this cohort with 95.2% sensitivity and 95.6% specificity (Fig. 4C). However, total plasma EV numbers and mean plasma EV size had lower accuracy profiles to predict preeclampsia (Fig. 4C). Calculated thresholds along with sensitivity and specificity are represented (Table 2). Threshold was selected using maximum likelihood ratio, and the corresponding row is bolded. Also, a separate summary table is provided highlighting threshold as selected by maximum likelihood ratio, ROC-AUC, p value, sensitivity, specificity, PPV, NPV and numbers of patients and controls, respectively. Collectively, this data suggests that quantitative syncytiotrophoblast EV profiling, but not whole plasma EV profiling, might serve as a noninvasive diagnostic in conditions of placental pathology such as preeclampsia.

**Table 2:** Youden's Index for the performance of the STEV platform in predicting preeclampsia in the presented cohort. STEV signal threshold cut-off of <0.316 was 95.2% sensitive and 95.6% specific for diagnosis of preeclampsia.

Threshold	Sensitivity%	95% CI		Specificity%		95% CI			Likelihood ratio	
<0.008	4.762	0.120% to 23.816%		100		85.181% to 100%				
<0.029	9.524	1.175% to 30.377%		100		85.181% to 100%				
<0.055	14.286	3.049% to 36.342%		100		85.181% to 100%				
<0.080	19.048	5.446% to 41.907%		100		85.181% to 100%				
<0.092	23.810	8.218% to 47.166%		100		85.181% to 100%				
<0.098	28.571	11.281% to 52.175%		100		85.181% to 100%				
<0.102	33.333	14.588% to 56.968%		100		85.181% to 100%				
<0.116	38.095	18.107% to 61.565%		100		85.181% to 100%				
<0.130	42.857	21.820% to 65.979%		100		85.181% to 100%				
<0.132	47.619	25.713% to 70.219%		100		85.181% to 100%				
<0.134	52.381	29.781% to 74.287%		100		85.181% to 100%				
<0.137	57.143	34.021% to 78.180%		100		85.181% to 100%				
<0.140	61.905	38.435% to 81.893%		100		85.181% to 100%				
<0.152	66.667	43.032% to 85.412%		100		85.181% to 100%				
<0.165	71.429	47.825% to 88.719%		100		85.181% to 100%				
<0.177	71.429	47.825% to 88.719%		95.652		78.051% to 99.890%			16.429	
<0.194	76.190	52.834% to 91.782%		95.652		78.051% to 99.890%			17.524	
<0.221	80.952	58.093% to 94.554%		95.652		78.051% to 99.890%			18.619	
<0.267	85.714	63.658% to 96.951%		95.652		78.051% to 99.890%			19.714	
<0.305	90.476	69.623% to 98.825%		95.652		78.051% to 99.890%			20.810	
**<0.316**	**95.238**	**76.184% to 99.880%**		**95.652**		**78.051% to 99.890%**			**21.905**	
<0.333	95.238	76.184% to 99.880%		91.304		71.962% to 98.929%			10.952	
<0.355	95.238	76.184% to 99.880%		86.957		66.411% to 97.225%			7.302	
<0.361	95.238	76.184% to 99.880%		82.609		61.219% to 95.049%			5.476	
<0.364	95.238	76.184% to 99.880%		78.261		56.297% to 92.540%			4.381	
<0.375	95.238	76.184% to 99.880%		73.913		51.595% to 89.771%			3.651	
<0.404	95.238	76.184% to 99.880%		69.565		47.081% to 86.790%			3.129	
<0.455	100	83.890% to 100%		69.565		47.081% to 86.790%			3.286	
<0.488	100	83.890% to 100%		65.217		42.734% to 83.624%			2.875	
<0.491	100	83.890% to 100%		60.870		38.542% to 80.292%			2.556	
<0.496	100	83.890% to 100%		56.522		34.495% to 76.809%			2.300	
<0.505	100	83.890% to 100%		52.174		30.588% to 73.180%			2.091	
<0.543	100	83.890% to 100%		47.826		26.820% to 69.412%			1.917	
<0.575	100	83.890% to 100%		43.478		23.191% to 65.505%			1.769	
<0.588	100	83.890% to 100%		39.130		19.708% to 61.458%			1.643	
<0.624	100	83.890% to 100%		34.783		16.376% to 57.266%			1.533	
<0.650	100	83.890% to 100%		30.435		13.210% to 52.919%			1.438	
<0.682	100	83.890% to 100%		26.087		10.229% to 48.405%			1.353	
<0.714	100	83.890% to 100%		21.739		7.460% to 43.703%			1.278	
<0.720	100	83.890% to 100%		17.391		4.951% to 38.781%			1.211	
<0.734	100	83.890% to 100%		13.043		2.775% to 33.589%			1.150	
<0.760	100	83.890% to 100%		8.696		1.071% to 28.0380%			1.095	
<0.797	100	83.890% to 100%		4.348		0.110% to 21.949%			1.045	
**Threshold**	**ROC-AUC**	**P Value**	**Sensitivity**	**Specificity**	**PPV**		**NPV**	**Patients**		**Controls**
<0.316	0.975	<0.001	95.2%	95.7%	95.3%		95.6%	n = 21		n = 23

## Discussion

Preeclampsia is one of the most common pregnancy-associated disorders, with increased morbidity and mortality risk rendered to the mother and the fetus. The diagnosis of this condition is based on clinical factors and typically occurs during the second trimester of pregnancy, but studies suggest that the mechanisms underlying the pathophysiology of preeclampsia are initiated early during the first trimester. Currently, there are no biomarkers for accurate and early diagnosis and monitoring of preeclampsia. Early diagnosis would enable closer monitoring and treatment of this condition, and may open the window for earlier intervention that might help reduce the associated risks to the fetus and the mother. In addition to investigations studying circulating free proteins and microRNAs for their biomarker potential, plasma extracellular vesicles, including exosomes, have been extensively studied in many medical fields for their diagnostic potential^38–50^.

Circulating EVs quantitative and cargo profiles are dynamic and may reflect condition-specific changes mediated upon their tissue of origin. In support of this concept, we recently showed that in the setting of cell/ organ transplantation, donor tissue specific exosome profiles accurately reflect injury inflicted from immunologic rejection on the transplant tissue in a time-specific manner^34,35^. These investigations led us to propose that placental pathology/ injury contributing to onset of preeclampsia during pregnancy might also lead to marked changes in placental tissue-specific EV profiles in maternal plasma. We report our initial investigation of this concept.

The findings of our investigation support the growing body of literature that pregnancy renders an altered EV profile status in the maternal plasma and that the fetal-derived syncytiotrophoblasts release EVs into the maternal plasma. Furthermore, conditions associated with placental pathology such as preeclampsia lead to differences in total EV quantities in maternal plasma, and more importantly significant changes in syncytiotrophoblast specific EVs may be observed. In the two study cohorts, we noted a trend towards increasing total EV quantities in maternal plasma with preeclampsia. A recent study by Salomon *et al*.^28^ showed that increasing EV quantities are noted during latter stages of gestation, and furthermore, significantly higher number of EVs were seen in preeclampsia subjects at each gestational stage (early: 11–14 weeks, mid: 22–28 weeks, late: 32–38 weeks). Analysis of plasma EV protein content for PLAP by enzyme linked immunoabsorbent assay showed that significantly higher PLAP content was seen in preeclampsia subjects overall. In gestation matched samples, however, when the PLAP content was normalized to total plasma EV quantity, the difference in PLAP content between preeclampsia and control subjects was diminished. For early gestational stage samples, the positive and negative predictive values by receiver operating characteristic curve for exosomal PLAP content was 75% and 76% respectively in this study.

In another study analyzing third trimester maternal plasma EV samples comparing preeclampsia subjects to normal controls, total plasma EV quantities were significantly increased in preeclampsia subjects^27^, with much higher fold differences in total plasma EV concentrations than that reported by Salomon *et al*.^28^. In early onset preeclampsia subjects compared to normotensive subjects <33-week gestation, 14.3-fold difference was seen in total plasma EV concentrations. In our investigation, we did not find such a big difference in total plasma EV numbers between normal and preeclampsia subjects, although our analysis was performed in plasma samples from pregnant females in 2^nd^ or 3^rd^ trimester of gestation. Our findings are more in accordance with Salomon *et al*.^28^, where there was <2-fold difference in total EV numbers between the two cohorts, with higher numbers seen in preeclampsia subjects. Pillay *et al*.^27^. also studied PLAP protein content in plasma EV fraction, and found increasing total PLAP content with early onset preeclampsia but not late onset preeclampsia. But when normalized to plasma EV quantities, the PLAP content to EV quantity ratio showed significantly decreased values in late onset preeclampsia subjects compared to normotensive controls. Both these studies utilized plasma PLAP protein content as a marker of placental EVs and did not directly measure PLAP surface expression on EVs. These studies suggest that preeclampsia is associated with a state of increased total EV quantities in maternal plasma but not necessarily increased syncytiotrophoblast EVs. Also, increased circulating quantity of placental markers such as PLAP might not necessarily translate to increased syncytiotrophoblast specific EV concentration in maternal plasma.

In support of the above idea, Tannetta *et al*.^51^ studied microvesicles released by perfused placentas in an *in vitro* closed-circuit system obtained by cesarean section from normal versus preeclampsia subjects. Unlike other studies that analyzed PLAP protein content by enzyme linked immunoabsorbent assay as a surrogate for placenta specific EVs, this study measured surface expression of PLAP on microvesicles. In flow cytometry analysis of microvesicles in the 300 nm to 1 μm range, they noted decreased percentage of PLAP positive microvesicles in perfused placentas from preeclampsia subjects compared to normal controls. In addition, mean fluorescence intensity for PLAP by flow cytometry was also decreased in microvesicles from preeclampsia subjects, suggesting that PLAP expressing EV quantity and the amount of PLAP carried on each microvesicle surface was decreased in microvesicles released by placentas from preeclampsia subjects. Accordingly, densitometry analysis of microvesicle protein content by Western blot for PLAP was significantly decreased in preeclampsia samples compared to normal controls. Furthermore, size distribution analysis of microvesicles by nanoparticle detector analysis, which enables analysis of particles in 10 nm to 1000 nm range, showed that placentas perfused from preeclampsia subjects contained fewer microvesicles in the exosome size range compared to placentas from normal subjects. Taken together, these results suggest that perfused placentas from preeclampsia subjects release decreased PLAP positive microvesicles, with lower PLAP surface expression per microvesicle and decreased microvesicles in the exosome range. Therefore, placental pathology associated with preeclampsia might manifest as a decrease in EV production with decreased expression of placenta specific marker on syncytiotrophoblast microvesicles, especially exosomes. The results of our investigation support these findings observed in a placenta perfusion model. Even though we found a general trend towards higher numbers of EVs in preeclampsia subjects, we noted that there was a significant reduction in syncytiotrophoblast specific EV signal in preeclampsia maternal plasma samples. This decrease in syncytiotrophoblast specific EV signal might be a reflection of lower number of placenta specific EVs, lower amounts of placenta specific marker on EVs, or a combination of both. These findings are in accordance to our proposed concept that tissue injury leads to decreased production of tissue specific EVs, especially exosomes.

In addition to PLAP, which is the most studied protein as a placenta specific marker, syncytin-1 and syncytin-2 proteins have also been reported to be altered in placental tissues from preeclampsia subjects^32,52^. Syncytin-1 and -2 are human endogenous retrovirus envelope proteins shown to play a critical role in trophoblast fusion, a process vital to formation of multinucleated syncytiotrophoblasts. Reduced expression of both syncytin-1 and syncytin-2 mRNA was shown in primary trophoblasts cultured from placentas of mothers with preeclampsia compared to normal controls. By Western blot analysis, decreased protein levels of syncytin-1 were noted in trophoblasts from subjects with severe preeclampsia, but a more pronounced difference in protein expression was noted with syncytin-2. The same group also analyzed EVs from maternal serum isolated using a polymer-based EV isolation kit. Densitometry analysis of serum EV protein content by Western blot showed no significant differences in syncytin-1 levels between control and preeclampsia samples, but syncytin-2 levels were significantly decreased in preeclampsia samples. These findings suggest that unlike PLAP, which has been shown to be increased in overall content in plasma EV fractions from preeclampsia subjects, placenta specific proteins syncytin-1 and syncytin-2 are decreased in the plasma extracellular vesicle pool of preeclampsia subjects.

It is important to note that decreased syncytin-1 EV signal in preeclampsia subjects detected by nanoparticle detector analysis may not necessarily mean that there is decreased production of EVs by syncytiotrophoblasts under this condition. It could very well be that in preeclampsia, the density of syncytin-1 surface expression on EVs is reduced, which could result in decreased synctin-1 EV signal detection without a decrease in overall EV production by syncytiotrophoblasts. Accordingly, just because our group and others have found increased EV quantities in maternal plasma from preeclampsia subjects does not mean that these increased numbers are due to increased EV production by placental syncytiotrophoblasts. At this point, our findings and the current literature support that overall a higher number of EVs are detected in maternal plasma from preeclampsia subjects compared to normal pregnancy. Also, there is evidence that increasing numbers of EVs are seen in maternal plasma during early to late gestation. Therefore, interpretation of the diagnostic potential of syncytiotrophoblast EVs must be investigated taking into consideration changes in peripheral blood EV profiles in a gestation-specific and condition-specific manner. To this effect, it will be important to understand gestation-specific changes in STEVs during normal pregnancy, before understanding specific changes associated with preeclampsia. Limitation of these findings is that the results in the current study were not confirmed in another cohort study. Lastly, current literature on EVs in the obstetrics diagnostics space reflects studies utilizing different EV isolation techniques, and various methods of signal quantitation. To carefully understand the potential of plasma EVs profiling for noninvasive diagnosis of placental pathologies, it will be important to standardize these methodologies and quantitation assays so that internal and intergroup variability is minimized.

## Conclusion

In summary, we performed a retrospective blinded analysis of maternal plasma EVs from gestation-matched subjects with preeclampsia versus normal pregnancy for characterization of syncytiotrophoblast-specific EV profiles. Utilizing placenta-specific protein, syncytin-1, as a surface marker on syncytiotrophoblast EVs, we found that syncytin-1 specific EV signal in maternal plasma is significantly decreased in subjects with preeclampsia. Further characterization of maternal plasma syncytin-1 specific EVs may enable the development of a noninvasive biomarker platform for diagnosis and monitoring of preeclampsia and other placental pathologies. Furthermore, characterization of circulating syncytiotrophoblast EV protein and nucleic acid cargoes may provide a mechanistic window into placental pathologies.

## Data Availability

All data generated or analyzed during this study are included in this published article.
